# An eigenvalue transformation technique for predicting drug-target interaction

**DOI:** 10.1038/srep13867

**Published:** 2015-09-09

**Authors:** Qifan Kuang, Xin Xu, Rong Li, Yongcheng Dong, Yan Li, Ziyan Huang, Yizhou Li, Menglong Li

**Affiliations:** 1 College of Chemistry, Sichuan University, Chengdu, 610064, China; 2 College of Computer Science, Sichuan University, Chengdu, 610064, China; 3 College of Life Science, Sichuan University, Chengdu, 610064, China

## Abstract

The prediction of drug-target interactions is a key step in the drug discovery process, which serves to identify new drugs or novel targets for existing drugs. However, experimental methods for predicting drug-target interactions are expensive and time-consuming. Therefore, the *in silico* prediction of drug-target interactions has recently attracted increasing attention. In this study, we propose an eigenvalue transformation technique and apply this technique to two representative algorithms, the Regularized Least Squares classifier (RLS) and the semi-supervised link prediction classifier (SLP), that have been used to predict drug-target interaction. The results of computational experiments with these techniques show that algorithms including eigenvalue transformation achieved better performance on drug-target interaction prediction than did the original algorithms. These findings show that eigenvalue transformation is an efficient technique for improving the performance of methods for predicting drug-target interactions. We further show that, in theory, eigenvalue transformation can be viewed as a feature transformation on the kernel matrix. Accordingly, although we only apply this technique to two algorithms in the current study, eigenvalue transformation also has the potential to be applied to other algorithms based on kernels.

The prediction of drug-target interactions is of key importance for the identification of new drugs or novel targets for existing drugs. However, validating drug targets by experiments is expensive and time-consuming. This consideration motivates the need to develop computational methods to predict drug-target interactions with high accuracy^1^.

Machine learning methods have recently been used to predict drug-target interactions. In general, this problem can be viewed as a link prediction problem. Based on the principle that similar drugs tend to have similar targets, many state-of-the-art methods have been proposed^1^,^2^,^3^,^4^,^5^,^6^,^7^,^8^,^9^,^10^,^11^,^12^,^13^. Among these methods, using kernels to incorporate multiple sources of information has proved efficient and popular^1^,^2^,^7^.

In this study, we propose an eigenvalue transformation technique and apply this technique to two representative algorithms based on kernels (RLS and SLP). The experimental results show that algorithms to which eigenvalue transformation is applied achieved better performance than the original algorithms on drug-target interaction prediction, i.e., eigenvalue transformation is an efficient technique for improving performance in predicting drug-target interactions. In a theoretical context, we further show that eigenvalue transformation can be viewed as a feature transformation on the kernel matrix. Thus, although we only apply this technique to two algorithms in this study, eigenvalue transformation has the potential to apply to other algorithms based on kernels. In addition, we investigate how eigenvalue transformation influences algorithms, and several interesting results are presented.

## Materials and Methods

### Materials

**Figure 1:**
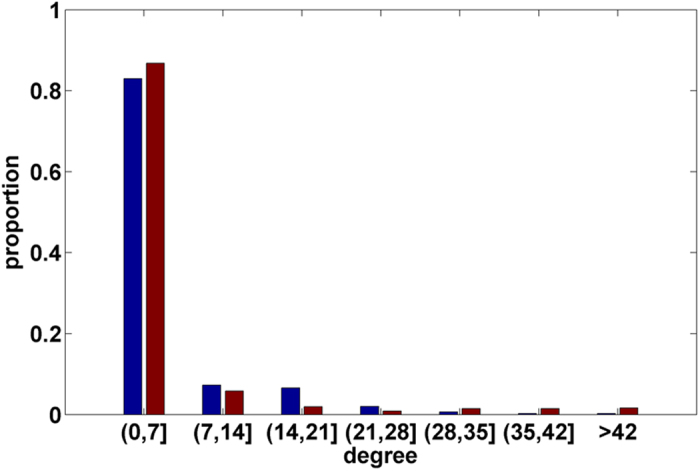
Degree distribution of drug and target. The blue bars indicate the degree distribution of the drug, and the red bars indicate the degree distribution of the target.

The known drug-target interaction network was obtained from DrugBank^14^. We extracted drugs that were (a) FDA approved, (b) with at least one ATC code^15^ and (c) with chemical structure information recorded in the KEGG database^16^. Ultimately, there were 3681 known drug-target interactions for 786 drugs and 809 targets. Figure 1 shows the degree distribution of drugs and targets.

Drug-ATC code interactions were retrieved from the KEGG database. The chemical structures of the drugs were derived from the DRUG and COMPOUND sections in the KEGG LIGAND database. Amino acid sequences of the target proteins were obtained from the UniProt database^17^.

### Problem formalization

We consider the problem of predicting new interactions in a drug-target interaction network. Formally, 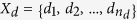 and 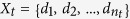 represent the set of drug nodes and the set of target nodes, respectively. The edges in the network are considered to represent the known drug-target interactions. The drug-target interaction network is characterized as an *n*_*d*_ × *n*_*t*_ adjacency matrix *Y*. That is, [*Y*]_*ij*_ = 1 if drug *d*_*i*_ interacts with target *t*_*j*_, and [*Y*]_*ij*_ = 0 otherwise. One of the main tasks of this study is to compute the prediction score of each non-interacting drug-target pair and then to predict new interactions among these non-interacting drug-target pairs.

### Model features

Three types of drug or target similarity matrices are employed in this study. The similarity between the chemical structures of drugs was computed using SIMCOMP^18^, resulting in a drug similarity matrix denoted by *S*_*chem*_. The ATC taxonomy similarity between drugs was computed using a semantic similarity algorithm^19^, resulting in another drug similarity matrix denoted by *S*_*ATC*_. The sequence similarity between targets was computed using a normalized version of the Smith-Waterman Score^20^, and this resulted in a target similarity matrix denoted by *S*_*seq*_. Finally, each similarity matrix was normalized as follows: 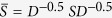; here, 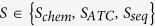, and for *S*, a diagonal matrix *D* was defined such that [*D*]_*ii*_ was the sum of row i of *S*. To satisfy the kernel matrices in a later algorithm, one should note that before being normalized, each similarity matrix has to be transformed to a symmetric and positive semi-definite matrix (adding the transpose and dividing by 2, then adding a proper positive real number multiple of the identity matrix to their diagonal^2^).

### Algorithms

In this study, we used two representative algorithms – the Regularized Least Squares classifier (RLS)^1^,^21^,^22^ and the semi-supervised Link Prediction classifier (SLP)^7^,^10^,^22^ to construct prediction models. These algorithms have shown good performance in predicting drug-target interactions. We briefly discuss these algorithms below.

### RLS

RLS is a basic supervised learning algorithm. If an appropriate kernel has been chosen for RLS, the accuracy of RLS will be similar to that of the support vector machine (SVM) method^23^, whereas the computational complexity of the RLS is much less than that of the SVM^21^. The general objective function of RLS is as follows:
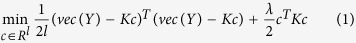


Here, *K* is a kernel matrix and *λ* is a regularization parameter. By taking the first derivative of *c*, the optimal solution regarding *c* is obtained: 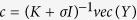, where *σ* = *λ1*. *I* is the identity matrix. Finally, the prediction score matrix 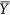 is computed as follows:
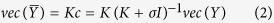


The RLS algorithm can be divided into three independent sub-algorithms for defining the kernel matrix: RLS-KP, RLS-KS and RLS-avg. Here, KP and KS denote Kronecker product^24^ and Kronecker sum^24^, respectively (more detailed descriptions of these sub-algorithms are provided in the Supplementary Algorithm).

### SLP

SLP is a semi-supervised learning algorithm^7^,^10^, and the basic assumption of SLP is that “two node pairs that are similar to each other are likely to have the same link strength”^7^. Based on this assumption, the general objective function of SLP is defined as follows:
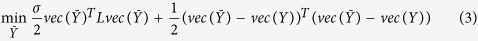
where *σ* is a regularization parameter and the Laplacian matrix 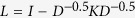; here, *D* is a diagonal matrix whose diagonal elements are 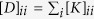. Finally, the prediction score matrix 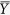 is computed as follows:
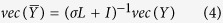


The SLP algorithm can also be divided into three independent sub-algorithms for defining the kernel matrix: SLP-KP, SLP-KS and SLP-avg. (More detailed descriptions of these sub-algorithms are provided in the Supplementary Algorithm).

### Algorithm with eigenvalue transformation applied

In this study, we apply an eigenvalue transformation technique to RLS and SLP. We briefly describe this technique as follows.

### Eigenvalue transformation in RLS

Let 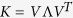 be the eigendecomposition of the kernel matrix *K* in RLS. Hence,
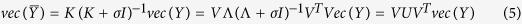
where *U* is a diagonal matrix whose diagonal elements are 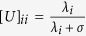 and *λ*_*i*_ is an eigenvalue of *K*. Here, we define a simple eigenvalue transformation as follows:
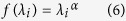
where *α* > 0 and *λ*_*i*_ ≥ 0; hence, this transformation is always well defined. We then substitute *f* (*λ*_*i*_) for *λ*_*i*_ in the equation for 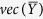. Finally, the solution of the equation specifying the prediction score matrix 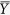 is as follows:
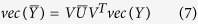


Here, 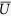 is a diagonal matrix whose diagonal elements are 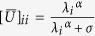 (detailed descriptions of the eigenvalue transformation applied in each sub-RLS algorithm are provided in the Supplementary Algorithm).

### Eigenvalue transformation in SLP

In SLP, *K* is a kernel matrix, and it is straightforward to show that 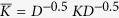 is also a kernel matrix. Let 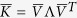 be the eigendecomposition of kernel matrix 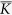 in SLP. Then,
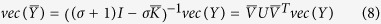
where *U* is a diagonal matrix whose diagonal elements are 
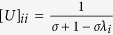
 and *λ*_*i*_ is an eigenvalue of 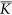. In an approach similar to that used with RLS, we apply the eigenvalue transformation to SLP. The solution is as follows:
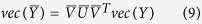


Here, 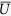 is a diagonal matrix whose diagonal elements are 
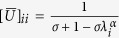
 (detailed descriptions regarding the eigenvalue transformation applied in each sub-SLP algorithm are provided in the Supplementary Algorithm).

### The mathematical meanings of eigenvalue transformation

We will now show that an eigenvalue transformation is equivalent to a mathematical transformation of the kernel matrix. To obtain a convenient framework for later description, we first extend the notion of kernel matrix power as follows:
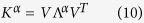


Here, *K* is the kernel matrix, 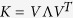 is the eigendecomposition of *K*, and *α* is a positive real number. It is straightforward to show that if *α* is an integer, Equation (10) is equivalent to the original matrix power. Based on this extended notion of kernel matrix power, the solution for the prediction score matrix 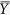 for the eigenvalue transformation applied to RLS can be rewritten as follows:
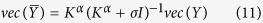


For the eigenvalue transformation applied to SLP, the solution for the prediction score matrix can be rewritten as follows:
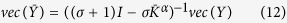


A comparison with the original RLS or SLP shows that the eigenvalue transformation applied to each algorithm is equivalent to a power transformation of the kernel matrix. Additionally, the kernel matrix is constructed from the drug or target similarity matrix for the purposes of this study. Therefore, the eigenvalue transformation could be considered a particular case of a feature transformation.

### Effect of eigenvalue exponent

We will now investigate the influence of the eigenvalue exponent on the algorithm. First, it is straightforward to show that Equation (11) and Equation (12) can be combined as follows:
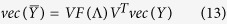


Here, 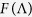 is a diagonal matrix whose diagonal elements are 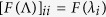. For RLS with the eigenvalue transformation applied, 
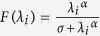
; for SLP with the eigenvalue transformation applied, 
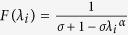
. Additionally, Equation (13) can be transformed as follows:
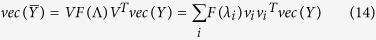


Here, *v*_*i*_ is the i-th column vector of *V*. We now normalize the prediction score as follows:
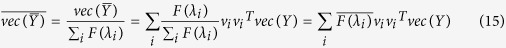


It is straightforward to prove that the normalized prediction score will not change the algorithm’s performance, so we need only investigate how the eigenvalue exponent influences the normalized prediction score. Note that we assume 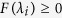 (it is straightforward to validate that RLS and SLP meet this assumption). Then, 
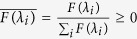
, 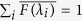. Therefore, the normalized prediction score 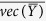 can be viewed as the weighted sum of 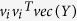, whose weighted coefficient is 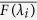. Here, 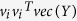 is determined by the drug or target similarity matrix and known drug-target interactions. Therefore, the eigenvalue exponent influences the normalized prediction score by adjusting the weight coefficient 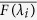. This argument conveys the mathematical essence of the influence of the eigenvalue exponent on the algorithm. In particular, under certain constraint conditions, for RLS, if the eigenvalue exponent decreases, the weighted coefficient 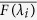 corresponding to a large eigenvalue *λ*_*i*_ will also decrease, whereas the weighted coefficient 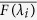 corresponding to a small eigenvalue *λ*_*j*_ will increase. This interesting result can be proven rigorously. A detailed proof is given in the Supplementary Effect of eigenvalue exponent on RLS.

**Data Accession**. Software and experimental data are available at: http://pan.baidu.com/s/1dDqDLuD

## Results and Discussion

### Evaluation

To compare the performance of the algorithms that included eigenvalue transformation with the original algorithms, simulation experiments were performed, all with 10-fold cross validation. For 10-fold cross validation, known drug-target interactions and unknown drug-target interactions were each randomly divided into 10 subsamples (“folds”) of roughly equal size; in each run of the method, one fold of known drug-target interactions and one fold of unknown drug-target interactions were left out by setting their entries in the adjacency matrix *Y* to 0. We then attempted to recover their true labels using the remaining data.

For the RLS or SLP algorithm with eigenvalue transformation applied, if the regularization parameter *σ* is fixed, we can show that the object function of RLS or SLP can achieve the minimum value when the eigenvalue exponent *α* = 0 (a detailed proof is given in Supplementary Theorem 1.0). However, when the objective function of RLS or SLP achieved the minimum value, we could not guarantee that the models would generalize satisfactorily, i.e., when the eigenvalue exponent *α* = 0, the training models may be overfitted. To a certain extent, the eigenvalue exponent *α* is similar to the penalty factor C in SVM. In each model, the optimal *α* is associated with particular training samples and features (later modeling experiment results will also validate this conclusion). Hence, we used the grid research method (essentially a method of exhaustive analysis that operates by trying a series of *α* values) to obtain the optimal *α*. This method is commonly used in SVM to obtain the optimal penalty factor C. For simplicity, in this study, the eigenvalue exponent *α* was chosen to range from 0 to 2 with a step of 0.1. Note that when *α* = 1, the algorithms with the eigenvalue transformation applied are equal to the original algorithms. In addition, we have chosen the values for the regularization parameter *σ* in a non-informative way^1^. In particular, *σ* was set to 0.05 for all RLS sub-algorithms, and *σ* was set to 0.01 for all SLP sub-algorithms.

We assessed the performance of the algorithms with two common quantitative indexes: AUC^25^ and AUPR^5^. The value of AUC is determined from the area below a curve relating the proportion of the true positives to the proportion of false positives, whereas the value of AUPR is determined from the area below a curve relating precision to recall. To compare each model’s performance, we combined AUC and AUPR as follows: 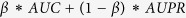; here, 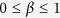. Strictly speaking, because there are few true drug-target interactions, the AUPR is a more meaningful quality measure than the AUC; therefore, 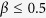. For simplicity, we selected 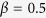 in this study.

**Figure 2:**
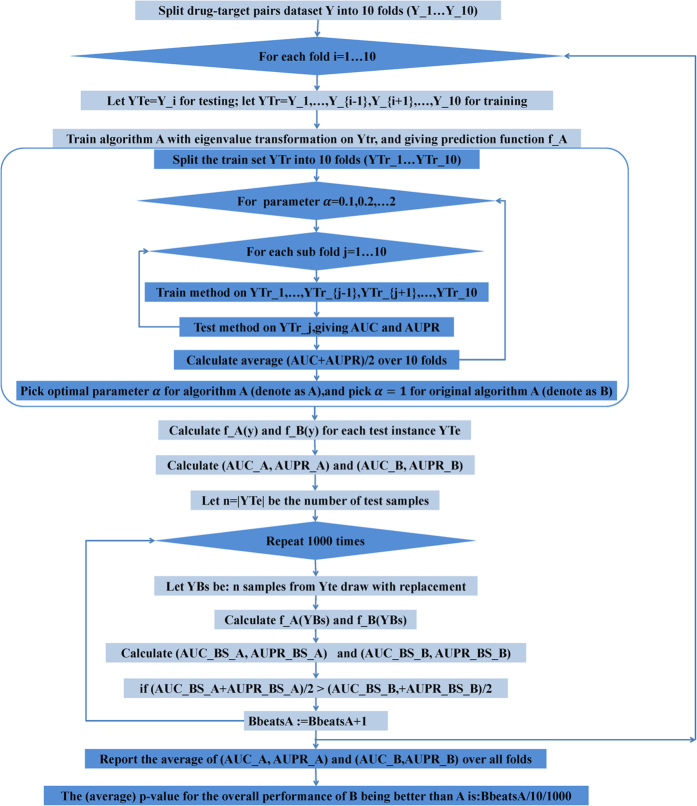
Overview of the main experiment workflow in this study. This workflow shows the main frame of double 10-fold cross validation. The inner cross validation procedure is used to obtain optimal parameter *α*, which is shown in the blue rectangle box. Algorithm A indicates algorithm with eigenvalue transformation applied, algorithm B indicates original algorithm. The p-value indicates the statistical significance of prediction performance by bootstrapping.

Intuitively, both algorithms and features will influence the *α*value tuning. The choice of different *α* values could be viewed as implementing different feature transformations on the kernel matrix. However, if the same feature transformations on the kernel matrix are applied to different algorithms, model performance may vary. Hence, the improvement in performance resulting from applying the transformation is dependent on the *α* value. However, we could not provide a satisfactory way to obtain an optimal *α* value, and it was necessary to try a series of different values. This difficulty represents an essential problem in model cross validation – it is trivially true that the AUC and/or AUPR are better with the eigenvalue transformation, as the original algorithm is just one particular case of the algorithm with the eigenvalue transformation applied, and the only way to prevent the problem is that *α* value selection must occur inside the algorithm. Hence, we used double cross validation for the eigenvalue transformation. The outer cross validation loop was used to estimate the model’s performance by predicting a ranking of one of the folds, using the rest as training data. As part of the training for each of the folds, another cross validation loop was used to select the value of *α*. In addition, to compute the statistical significance of prediction performance, we used bootstrapping to compute the AUC and AUPR for each model. Detailed illustration of the main workflow of above experiments has been shown in Fig. 2.

### Model performance

In the analyses performed in this study, each sub-algorithm needs two input similarity matrices *S*_*d*_ and *S*_*t*_. Here, for targets, *S*_*t*_ = *S*_*seq*_; for drugs, we used three types of similarity matrix: *S*_*d*_ = *S*_*chem*_, *S*_*d*_ = *S*_*ATC*_ and *S*_*d*_ = (*S*_*chem*_ + *S*_*ATC*_)/2. In the modeling experiment, Table 1 contains double 10-fold cross validation results for RLS-KP with the eigenvalue transformation applied when *S*_*d*_ = *S*_*chem*_ (more detailed results for other sub-algorithms can be found in Supplementary Tables S1–S17). According to the results, although, the optimal *α* may be different for different outer folds. However, the performance of each outer fold is consistent with the performance of the nine inner training folds. That is, to a certain degree, prediction models built by the sub-algorithm with the eigenvalue transformation applied could also achieve good performance on unseen data. In addition, there are four sub-algorithms (except RLS-KS and RLS-avg) with the eigenvalue transformation applied achieve better performance than the original sub-algorithms, i.e., the eigenvalue transformation is an efficient technique to improve the predictive performance of drug-target interaction models. And the performance of each prediction model built with the drug similarity matrix 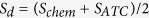 was always better than that of 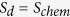 or 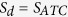, i.e., information on the drug chemical structure and the drug ATC code is complementary in the prediction of drug-target interactions. In addition, according to results, it seems to be against common sense that the AUC and AUPR are higher on the test set than on the training set when inner 10 fold cross validation was performed. We think the abnormal performance is due to the samples (known drug-target pairs) involved in model training. When outer 10-fold cross validation was performed, 90% known drug-target pairs were used as positive samples in each iteration for model training. For inner 10-fold cross validation, this number would be ~81% (0.9*0.9).

### New prediction

To analyze the practical relevance of the eigenvalue transformation technique for predicting novel drug-target interactions, we reconstructed the model with all known drug-target interactions and ranked the non-interacting pairs according to the prediction scores. We estimated that the most highly ranked drug-target pairs were most likely to be potential interactions. Here, the prediction model built by RLS-KP 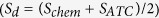 And a list of the top 15 new interactions predicted by RLS-KP with the eigenvalue transformation applied (*α* = 0.4) can be found in Table 2. To facilitate benchmark comparisons, a list of the top 15 new interactions predicted by the original RLS-KS (*α* = 1) is also shown in Table 3. Strictly speaking, for each non-interacting pair, we could not be entirely sure that this pair is truly a non-interaction pair in the real world, even it had a low prediction score in the computational model. The experimental facilities needed to validate each non-interaction pair were lacking. Therefore, we used a practical but not strictly correct way to validate the non-interaction pairs. This approach has been widely used in similar areas of study^1^,^11^. We validated each set of 15 top-ranking non-interaction pairs by researching whether this pair had been recorded as an interaction pair in the Kegg, ChEMBL^26^ or SuperTarget^27^ database. According to Table 2 and Table 3, in the top 15 new interactions, three interactions predicted by the original RLS-KP could be found in the KEGG database, whereas five interactions predicted by RLS-KP with the eigenvalue transformation applied could be found in the KEGG database. Additionally, these three validated interactions predicted by the original RLS-KP were among the five validated interactions predicted by the RLS-KP with the eigenvalue transformation applied. Accordingly, the eigenvalue transformation technique is practically relevant for predicting novel drug-target interactions.

## Conclusions

We presented an eigenvalue transformation technique and applied the technique to two representative algorithms. The performance of the algorithms with the eigenvalue transformation applied was better than that of the corresponding original algorithms. The experimental results show that the eigenvalue transformation technique is a simple but efficient method to improve the performance of algorithms used to predict drug-target interactions. A further theoretical analysis of eigenvalue transformation showed that eigenvalue transformation could be viewed as a particular feature transformation on the kernel matrix. In addition, the influence of the eigenvalue exponent on the algorithm was investigated, and several interesting results were obtained.

As an eigenvalue transformation can be viewed as a particular feature transformation on a kernel matrix, the eigenvalue transformation can potentially be applied to other algorithms based on a kernel matrix (such as SVM). The eigenvalue transformation has been shown to improve the performance of algorithms used to predict drug-target interactions. Therefore, eigenvalue transformations also have the potential to be applied to other similar prediction systems, such as those used to predict drug-side effect associations.

## Additional Information

**How to cite this article**: Kuang, Q. *et al.* An eigenvalue transformation technique for predicting drug-target interaction. *Sci. Rep.* **5**, 13867; doi: 10.1038/srep13867 (2015).

## Supplementary Material

Supplementary Information

## Figures and Tables

**Table 1:** Performance of RLS-KP by 10-fold cross validation with *S*_*d*_ = *S*_*chem*_.

**Fold Id**	**Original algorithm**				**Algorithm with eigenvalue transformation applied**					**p**
	**Training set**		**Test set**		**Training set**		**Test set**		***α***	
	**AUC**	**AUPR**	**AUC**	**AUPR**	**AUC**	**AUPR**	**AUC**	**AUPR**		
1	93.0	42.9	92.8	51.2	93.5	56.7	94.3	68.9	0.5	0
2	92.5	42.2	92.7	52.9	93.7	56.8	93.7	66.5	0.6	0
3	92.8	42.9	92.7	50.0	93.5	57.4	93.3	65.9	0.5	0
4	92.3	43.3	93.3	44.9	93.6	58.0	93.9	63.7	0.5	0
5	92.6	43.0	92.8	49.5	93.4	57.5	94.8	68.6	0.5	0
6	92.4	43.0	93.0	44.0	93.4	57.6	94.0	60.1	0.6	0
7	92.7	44.0	92.9	42.4	93.6	58.6	94.1	62.9	0.5	0
8	92.4	43.6	91.9	44.5	93.5	58.9	93.6	59.4	0.5	0
9	92.5	42.9	93.8	50.2	93.7	57.3	94.5	65.3	0.5	0
10	92.2	43.1	94.3	51.9	93.4	57.0	94.4	68.1	0.5	0
average	92.5	43.1	93.0	48.1	93.5	57.6	94.1	65.0		0

The AUC scores and AUPR scores are normalized to 100. The p indicates p-value of bootstrapping.

**Table 2:** The top 15 new interactions predicted by RLS-KP with the eigenvalue transformation applied.

**Rank**	**Drug ID**	**New drug-target interactions**		**Target name**
		**Drug name**	**Target ID**	
**1**	**DB01544**	**Flunitrazepam**	**P14867**	**Gamma-aminobutyric acid receptor subunit alpha-1**
**2**	**DB01567**	**Fludiazepam**	**Q16445**	**Gamma-aminobutyric acid receptor subunit alpha-6**
3	DB00546	Adinazolam	Q16445	Gamma-aminobutyric acid receptor subunit alpha-6
**4**	**DB01567**	**Fludiazepam**	**P48169**	**Gamma-aminobutyric acid receptor subunit alpha-4**
5	DB00546	Adinazolam	P48169	Gamma-aminobutyric acid receptor subunit alpha-4
**6**	**DB01567**	**Fludiazepam**	**Q9UN88**	**Gamma-aminobutyric acid receptor subunit theta**
7	DB01394	Colchicine	P68371	Tubulin beta-4B chain
8	DB00334	Olanzapine	P30939	5-hydroxytryptamine receptor 1F
9	DB00546	Adinazolam	Q9UN88	Gamma-aminobutyric acid receptor subunit theta
10	DB00408	Loxapine	P30939	5-hydroxytryptamine receptor 1F
**11**	**DB00909**	**Zonisamide**	**Q9UQD0**	**Sodium channel protein type 8 subunit alpha**
12	DB01586	Ursodeoxycholic acid	Q04828	Aldo-keto reductase family 1 member C1
13	DB00936	Salicyclic acid	P52895	Aldo-keto reductase family 1 member C2
14	DB08901	Ponatinib	A9UF02	Non-specific protein-tyrosine kinase
15	DB06772	Cabazitaxel	Q71U36	Tubulin alpha-1A chain

Five interactions have been confirmed (shown in bold).

**Table 3:** The top 15 new interactions predicted by the original RLS-KP.

**Rank**	**New drug-target interactions**			
	**Drug ID**	**Drug name**	**Target ID**	**Target name**
1	DB00474	Methohexital	P47869	Gamma-aminobutyric acid receptor subunit alpha-2
2	DB00474	Methohexital	P31644	Gamma-aminobutyric acid receptor subunit alpha-5
**3**	**DB01567**	**Fludiazepam**	**Q16445**	**Gamma-aminobutyric acid receptor subunit alpha-6**
4	DB00474	Methohexital	P34903	Gamma-aminobutyric acid receptor subunit alpha-3
5	DB00546	Adinazolam	Q16445	Gamma-aminobutyric acid receptor subunit alpha-6
**6**	**DB01567**	**Fludiazepam**	**P48169**	**Gamma-aminobutyric acid receptor subunit alpha-4**
7	DB00474	Methohexital	Q16445	Gamma-aminobutyric acid receptor subunit alpha-6
8	DB00546	Adinazolam	P48169	Gamma-aminobutyric acid receptor subunit alpha-4
**9**	**DB01544**	**Flunitrazepam**	**P14867**	**Gamma-aminobutyric acid receptor subunit alpha-1**
10	DB00228	Enflurane	P23416	Glycine receptor subunit alpha-2
11	DB00228	Enflurane	O75311	Glycine receptor subunit alpha-3
12	DB00474	Methohexital	P48169	Gamma-aminobutyric acid receptor subunit alpha-4
13	DB00599	Thiopental	P18507	Gamma-aminobutyric acid receptor subunit gamma-2
14	DB01159	Halothane	P23416	Glycine receptor subunit alpha-2
15	DB00753	Isoflurane	P23416	Glycine receptor subunit alpha-2

Three interactions have been confirmed (shown in bold).
