# Supplementary material for: An eigenvalue transformation technique for predicting drug-target interaction
Source: Sci Rep. 2015 Sep 9;5:13867. doi: 10.1038/srep13867 (PMC4563363; doi:10.1038/srep13867)
Supplement: Supplementary Information [file srep13867-s1.pdf]

Supplementary Materials for

An eigenvalue transformation technique for predicting drug-target interaction

Qifan Kuang, Xin Xu, Rong Li, Yongcheng Dong, Yan Li, Ziyan Huang, Yizhou Li and Menglong Li

This PDF file includes:

Algorithms

Effect of eigenvalue exponent on RLS

Theorem 1.0

Table S1. Performance of RLS-KP by 10-fold cross validation with $S_d = S_{ATC}$

Table S2. Performance of RLS-KP by 10-fold cross validation with $S_d = \frac{S_{chem} + S_{ATC}}{2}$

Table S3. Performance of RLS-KS by 10-fold cross validation with $S_d = S_{chem}$

Table S4. Performance of RLS-KS by 10-fold cross validation with $S_d = S_{ATC}$

Table S5. Performance of RLS-KS by 10-fold cross validation with $S_d = \frac{S_{chem} + S_{ATC}}{2}$

Table S6. Performance of RLS-avg by 10-fold cross validation with $S_d = S_{chem}$

Table S7. Performance of RLS-avg by 10-fold cross validation with $S_d = S_{ATC}$

Table S8. Performance of RLS-avg by 10-fold cross validation with $S_d = \frac{S_{chem} + S_{ATC}}{2}$

Table S9. Performance of SLP-KP by 10-fold cross validation with $S_d = S_{chem}$

Table S10. Performance of SLP-KP by 10-fold cross validation with $S_d = S_{ATC}$

Table S11. Performance of SLP-KP by 10-fold cross validation with $S_d = \frac{S_{chem} + S_{ATC}}{2}$

Table S12. Performance of SLP-KS by 10-fold cross validation with $S_d = S_{chem}$

Table S13. Performance of SLP-KS by 10-fold cross validation with $S_d = S_{ATC}$

Table S14. Performance of SLP-KS by 10-fold cross validation with $S_d = \frac{S_{chem} + S_{ATC}}{2}$

Table S15. Performance of SLP-avg by 10-fold cross validation with $S_d = S_{chem}$

Table S16. Performance of SLP-avg by 10-fold cross validation with $S_d = S_{ATC}$

Table S17. Performance of SLP-avg by 10-fold cross validation with $S_d = \frac{S_{chem} + S_{ATC}}{2}$

Algorithms

RLS-KP

In RLS-KP, kernel matrix is defined as: $K = S_d \otimes S_t$, here S_d indicates drug similarity matrix, S_t indicates target similarity matrix and the $\otimes$ operator indicates the Kronecker Product. Based on RLS-KP, the coefficient c is obtained by minimizing the following objective function: $\min_{c \in R^l} \frac{1}{2l}(vec(Y^T) - Kc)^T(vec(Y^T) - Kc) + \frac{\lambda}{2}c^T Kc$. Here, λ is a regularization parameter. By taking the first derivative of c, the optimal solution of c is obtained: $c = (K + \sigma I)^{-1} vec(Y^T)$, where $\sigma = \lambda l$. And then, the prediction score matrix is computed as follows: $vec(\overline{Y}^T) = K(K + \sigma I)^{-1} vec(Y^T)$. In theory, $\overline{Y}$ could be directly calculated using above equation. However, for high dimension of K, huge memory overhead will be necessary for practice calculation. Therefore, several mathematical techniques are employed to reduce the computational cost. We briefly discuss this process as follows: Let $S_d = V_d \Lambda_d V_d^T$ and $S_t = V_t \Lambda_t V_t^T$ be the Eigen decompositions of these matrices. Then based on Kronecker Product property: $(AB) \otimes (CD) = (A \otimes C)(B \otimes D)$, we will have $K = S_d \otimes S_t = V \Lambda V^T$, where $V = V_d \otimes V_t$ and $\Lambda = \Lambda_d \otimes \Lambda_t$, and then we use a further property of Kronecker Product: $(A \otimes B) vec(C) = vec(BCA^T)$. Combining these facts, we could get the final solution as follows:

$$\overline{Y} = V_d Z^T V_t^T \qquad (1)$$

Where $vec(Z) = (\Lambda_d \otimes \Lambda_t)(\Lambda_d \otimes \Lambda_t + \sigma I)^{-1} vec(V_t^T Y^T V_d)$.

When RLS-KP is applied eigenvalue transformation, the solution of prediction score matrix will be written as follows:

$$\overline{Y} = V_d \overline{Z}^T V_t^T \qquad (2)$$

Where $vec(\overline{Z}) = (\Lambda_d^{\alpha} \otimes \Lambda_t^{\alpha})(\Lambda_d^{\alpha} \otimes \Lambda_t^{\alpha} + \sigma I)^{-1} vec(V_t^T Y^T V_d)$, here $\Lambda_d^{\alpha}(\Lambda_t^{\alpha})$ is diagonal matrix whose diagonal elements are: $[\Lambda_d^{\alpha}]_{ii} = \lambda_{d_i}^{\alpha}([\Lambda_t^{\alpha}]_{ii} = \lambda_{t_i}^{\alpha})$.

RLS-KS

In RLS-KS, kernel matrix is defined as: $K = S_d \oplus S_t$, here the $\oplus$ operator indicates the Kronecker Sum, and Kronecker Sum could be transformationed as Kronecker Product: $S_d \oplus S_t = I \otimes S_d + S_t \otimes I$. Based on RLS-KS, the coefficient c is obtained by minimizing the following objective function: $\min_{c \in R^l} \frac{1}{2l}(vec(Y) - Kc)^T(vec(Y) - Kc) + \frac{\lambda}{2}c^T Kc$. Similar to RLS-KP, we could get the final solution of $\overline{Y}$ as follows:

$$vec(\overline{Y}) = V_d Z V_t^T \qquad (3)$$

Where $vec(Z) = (\Lambda_d \oplus \Lambda_t)(\Lambda_d \oplus \Lambda_t + \sigma I)^{-1} vec(V_d^T Y V_t)$.

When RLS-KS is applied eigenvalue transformation, the solution of prediction score matrix will be written as follows:

$$vec(\overline{Y}) = V_d \overline{Z} V_t^T \qquad (4)$$

Where $vec(\overline{Z}) = (\Lambda_d \oplus \Lambda_t)^{\alpha}((\Lambda_d \oplus \Lambda_t)^{\alpha} + \sigma I)^{-1} vec(V_d^T Y V_t)$, here $(\Lambda_d \oplus \Lambda_t)^{\alpha}$ is diagonal matrix whose

diagonal elements belong to $\{(\lambda_{d_j} + \lambda_{t_i})^{\alpha}\}_{i,j}$.

RLS-avg

In RLS-avg, kernel matrix will be defined as: $K_d = S_d$ and $K_t = S_t$, respectively. That is, RLS-avg constructs two independent models, and the average of the prediction score matrices of these two models is then taken as the final prediction score matrix. Here, we illustrate the computational process of RLS-avg based on $K_d = S_d$. The coefficient C_d is obtained by minimizing the following objective function: $\min_{C_d} \frac{1}{2l}\|Y - K_d C_d\|_F^2 + \frac{\lambda}{2} C_d{}^T K_d C_d$. By taking first derivative of C_d , the optimal solution of C_d is obtained: $C_d = (K + \sigma I)^{-1} Y$, where $\sigma = \lambda l$. And then $\overline{Y}_d$ is computed as follows: $\overline{Y}_d = K_d (K_d + \sigma I)^{-1} Y$. Similarity, RLS-avg based on $K_t = S_t$, we could get the solution of $\overline{Y}_t$. The final prediction score matrix is as follows:

$$\overline{Y} = (\overline{Y}_d + \overline{Y}_t{}^T)/2 \qquad (5) .$$

When RLS-avg is applied eigenvalue transformation, the solution of prediction score matrix will be written as follows:

$$\overline{Y} = \frac{1}{2}(V_d \overline{U_d} V_d{}^T + V_t{}^T \overline{U_t} V_t) \qquad (6)$$

Here $S_d = V_d \Lambda_d V_d{}^T$ and $S_t = V_t \Lambda_t V_t^T$ are the Eigen decompositions of these two matrices, $\overline{U_d}$ is a diagonal matrix whose diagonal elements are: $[\overline{U_d}]_{ii} = \frac{\lambda_{b_i}{}^{\alpha}}{\sigma + \lambda_{b_i}{}^{\alpha}}$. The matrix $\overline{U_t}$ is defined similarly.

SLP-KP

In SLP-KP, the Laplacian matrix of SLP-KP is defined as: $L = I - S_t \otimes S_d$ (strictly speaking, Laplacian matrix should be written as follows: $L = I - (D_t{}^{-0.5} S_t D_t{}^{-0.5}) \otimes (D_d{}^{-0.5} S_d D_d{}^{-0.5})$, here D_d is a diagonal matrix whose diagonal elements are $[D_d]_{ii} = \sum_j [S_d]_{ij}$. The D_t is defined similarly. While, in data preprocessing, S_d and S_t have been normalized as: $\overline{S_d} = D_d{}^{-0.5} S_d D_d{}^{-0.5}$, $\overline{S_t} = D_t{}^{-0.5} S_t D_t{}^{-0.5}$. Hence, in this study, Laplacian matrix is defined as: $L = I - S_t \otimes S_d$). The objective function of SLP-KP is as follows: $\min_{\overline{Y}} \frac{\sigma}{2} vec(\overline{Y})^T L vec(\overline{Y}) + \frac{1}{2}\|vec(\overline{Y}) - vec(Y)\|_F^2$. By taking first derivative of $\overline{Y}$, the optimal solution of $\overline{Y}$ is obtained: $vec(\overline{Y}) = (\sigma L + I)^{-1} vec(Y)$. We use mathematical technique which is similar to RLS, and then the final prediction score matrix could be written as:

$$\overline{Y} = V_d (D * (V_d{}^T Y V_t)) V_t^T \qquad (7)$$

Where $[D]_{ij} = (1 + \sigma(1 - [\Lambda_d]_{ii}[\Lambda_t]_{jj}))^{-1}$, here $S_d = V_d \Lambda_d V_d{}^T$ and $S_t = V_t \Lambda_t V_t^T$ are the Eigen decompositions of these two matrices. In addition, the symbol $*$ indicates Hadamard product of matrices.

When SLP-KP is applied eigenvalue transformation, the solution of prediction score matrix will be written as follows:

$$\overline{Y} = V_d (\overline{U} * (V_d{}^T Y V_t)) V_t^T \qquad (8)$$

Where $[\overline{U}]_{ij} = (1+\sigma(1-\lambda_{d_i}{}^{\alpha}\lambda_{t_j}{}^{\alpha}))^{-1}$.

SLP-KS

In SLP-KS, the Laplacian matrix of SLP-KS is defined as: $L = I - S_t \oplus S_d$. The computational processing of SLP-KS is similar to SLP-KP, here we present the final solution as follows:

$$\overline{Y} = V_d(D*(V_d{}^T Y V_t))V_t^T \qquad (9)$$

Where $[D]_{ij} = (1+\sigma(3-[\Lambda_d]_{ii}-[\Lambda_t]_{jj}))^{-1}$.

When SLP-KS is applied eigenvalue transformation, the solution of prediction score matrix will be written as follows:

$$\overline{Y} = V_d(\overline{U}*(V_d{}^T Y V_t))V_t^T \qquad (10)$$

Where $[\overline{U}]_{ij} = (1+\sigma(3-(\lambda_{d_i}+\lambda_{t_j})^{\alpha}))^{-1}$.

SLP-avg

The overall handling of SLP-avg is similar to RLS-avg, in which SLP-avg will also construct two independent models. Here, we illustrate the computational process of SLP-avg based on drugs. The Laplacian matrix of SLP-avg is defined as: $L_d = I - S_d$. And the object function of SLP-avg is as follows: $\min_{\overline{Y}_d} \frac{\sigma}{2}\overline{Y}_d{}^T L \overline{Y}_d + \frac{1}{2}\left\|\overline{Y}_d - Y\right\|_F^2$. By taking first derivative of $\overline{Y}_d$, the optimal solution of $\overline{Y}_d$ is obtained: $\overline{Y}_d = (\sigma L_d + I)^{-1}Y$. Similarity, SLP-avg based on targets, we could get the solution of $\overline{Y}_t$. The final prediction score matrix is as follows:

$$\overline{Y} = (\overline{Y}_d + \overline{Y}_t)/2 \qquad (11)\ .$$

When SLP-avg is applied eigenvalue transformation, the solution of prediction score matrix will be written as follows:

$$\overline{Y} = \frac{1}{2}(V_d\overline{U_d}V_d{}^T + V_t{}^T\overline{U_t}V_t) \qquad (12)$$

Here, $S_d = V_d\Lambda_d V_d^T$ and $S_t = V_t\Lambda_t V_t^T$ are Eigen decompositions of these two matrices, $\overline{U_d}$ is a diagonal matrix whose diagonal elements are: $[\overline{U_d}]_{ii} = (1+\sigma(1-\lambda_{d_i}{}^{\alpha}))^{-1}$. The matrix $\overline{U_t}$ is defined similarly.

Effect of eigenvalue exponent on RLS

For RLS, under some constraint condition, if eigenvalue exponent is decreased, the weighted coefficient $\overline{F(\lambda_i)}$ corresponding to large eigenvalue λ_i will also be decreased, while the weighted coefficient $\overline{F(\lambda_j)}$ corresponding to small eigenvalue λ_j will be increased. Here, this interesting result regarding influence of eigenvalue exponent on RLS-KP will be proven in mathematical. Before we finish the proof of this result, we need to prove some lemmas.

Lemma 1. If M is a $n\times n$ matrix, for $i=1,2,...,n; j=1,2,...,n$, $[M]_{ij}\ge 0$ and $\sum_{j=1}^{n}[M]_{ij}=1$. Then eigenvalue λ of M will meet this constraint condition: $|\lambda|\le 1$.

Proof: $X=[x_1,x_2,...,x_n]^T$ indicates the Eigen vector corresponding to $|\lambda|$. We assume that $x_k=\max_{1\le i\le n}\{|x_i|\}$. Then $|\lambda x_k|=|\sum_{j=1}^{n}[M]_{kj}x_j|\le\sum_{j=1}^{n}|[M]_{kj}|\bullet|x_j|\le\sum_{j=1}^{n}|[M]_{kj}|\bullet|x_k|=(\sum_{j=1}^{n}|[M]_{kj}|)|x_k|=|x_k|$, hence $|\lambda|\le 1$. Moreover, we could validate $X=[1,1,...,1]^T$ is an Eigen vector of M corresponding to $\lambda=1$.

Lemma 2. If S is a positive semidefinite matrix and $[S]_{ij}\ge 0$. Then eigenvalue λ of $D^{-0.5}SD^{-0.5}$ will be meet this constraint condition: $0\le\lambda\le 1$. Here D is a diagonal matrix whose diagonal elements are: $[D]_{ii}=\sum_j[S]_{ij}$.

Proof: we assume that $\overline{S}=D^{-0.5}SD^{-0.5}$ and $\widehat{S}=D^{-1}S$. First, it's easy to validate that $[\widehat{S}]_{ij}\ge 0$ and $\sum_j[\widehat{S}]_{ij}=1$, according to Lemma 1, the eigenvalue $\widehat{\lambda}$ of $\widehat{S}$ will meet: $|\widehat{\lambda}|\le 1$. On the other hand, $\overline{S}=D^{0.5}\widehat{S}D^{-0.5}$, if $\widehat{V}$ is the Eigen vector of $\widehat{S}$ corresponding to $\widehat{\lambda}$, then $D^{0.5}\widehat{V}$ is the Eigen vector of $\overline{S}$ corresponding to eigenvalue $\widehat{\lambda}$. Hence, eigenvalue of $\widehat{S}$ is equal to $\overline{S}$, that is, eigenvalue λ of $\overline{S}$ will also meet: $|\lambda|\le 1$. Besides, it's also easy to validate that $\overline{S}$ is a positive semidefinite matrix, hence $0\le\lambda\le 1$

Lemma 3. If $f(b)>g(b)$, $f(a)<g(a)$ and $f''(x)<g''(x)$. Then in $x\in[a,b]$, $f(x)$ and $g(x)$ have one and only common solution.

Proof: we assume that $h(x)=f(x)-g(x)$, then in $x\in[a,b]$, $h(a)<0$ and $h(b)>0$, according to mathematical analysis, existing at least one $\overset{\frown}{x}\in[a,b]$ is the solution of $h(x)=0$. That is, $f(x)$ and $g(x)$ have at least one common solution. If $f(x)$ and $g(x)$ have more than 1 common solutions. We assume that $\{x_1,...,x_n\}$ is common solution set and $a<x_1<...<x_n<b$. Then $h(x_n)=0$ and $h(x_{n-1})=0$, according to mathematical analysis, existing at least one $\overline{x}\in[x_{n-1},x_n]$ is the solution of $h'(x)=0$, that is, $f'(\overline{x})=g'(\overline{x})$. On the other hand, in $x\in[a,b]$, $f''(x)<g''(x)$, hence in $x\in(x_n,b]$, $f'(x)<g'(x)$, then $\int_{x_n}^{b}f'(x)dx<\int_{x_n}^{b}g'(x)dx$, that is $f(b)<g(b)$ which is a contradiction (in condition, $f(b)>g(b)$). Combining these facts, in $x\in[a,b]$, $f(x)$ and $g(x)$ have one and only common solution.

In RLS-KP applied eigenvalue transformation, the weighted coefficient $\overline{F(\lambda_i)}=\frac{\lambda_i^{\alpha}/(\sigma+\lambda_i^{\alpha})}{\sum_j\lambda_j^{\alpha}/(\sigma+\lambda_j^{\alpha})}$, here λ_i is eigenvalue of kernel matrix K. In RLS-KP, $\lambda_i\in\{\lambda_{d_k}\lambda_{t_j}\}_{k,j}$, here λ_{d_k} and λ_{t_j} are

eigenvalues of S_d and S_t, respectively. According to Lemma 2, $0 \le \lambda_{d_k} \le 1$, $0 \le \lambda_{t_j} \le 1$, therefore, $0 \le \lambda_i \le 1$. On the other hand, we assume that $\{\lambda_1, ..., \lambda_n\}$ is the eigenvalue set of K and $0 \le \lambda_1 \le ... \le \lambda_n \le 1$ (Note that, in later proof, we only consider this case: $\lambda_1 > 0$). And in $\lambda \in [\lambda_1, \lambda_n]$, we define two functions of λ as follows: $f(\lambda) = \frac{\lambda^\alpha / (\sigma + \lambda^\alpha)}{\sum_{i=1}^n \lambda_i^\alpha / (\sigma + \lambda_i^\alpha)}$, $g(\lambda) = \frac{\lambda^\beta / (\sigma + \lambda^\beta)}{\sum_{i=1}^n \lambda_i^\beta / (\sigma + \lambda_i^\beta)}$, here $\alpha > \beta$. Now we'll prove that (i) $f(\lambda_n) > g(\lambda_n)$, $f(\lambda_1) < g(\lambda_1)$; (ii)if σ is small enough, then $f''(\lambda) < g''(\lambda)$.

Proof (i): here, we'll prove $f(\lambda_n) > g(\lambda_n)$ as follows (the proof of $f(\lambda_1) < g(\lambda_1)$ is similarly):

$$f(\lambda_n) > g(\lambda_n) \Leftrightarrow \frac{\lambda_n^\alpha / (\sigma + \lambda_n^\alpha)}{\sum_{i=1}^n \lambda_i^\alpha / (\sigma + \lambda_i^\alpha)} > \frac{\lambda_n^\beta / (\sigma + \lambda_n^\beta)}{\sum_{i=1}^n \lambda_i^\beta / (\sigma + \lambda_i^\beta)} \Leftrightarrow \sum_{i=1}^n \frac{\lambda_n^\alpha \lambda_i^\beta}{(\sigma + \lambda_n^\alpha)(\sigma + \lambda_i^\beta)} > \sum_{i=1}^n \frac{\lambda_n^\beta \lambda_i^\alpha}{(\sigma + \lambda_n^\beta)(\sigma + \lambda_i^\alpha)}$$

$$\Leftarrow \frac{\lambda_n^\alpha \lambda_i^\beta}{(\sigma + \lambda_n^\alpha)(\sigma + \lambda_i^\beta)} > \frac{\lambda_n^\beta \lambda_i^\alpha}{(\sigma + \lambda_n^\beta)(\sigma + \lambda_i^\alpha)} \Leftrightarrow \sigma^2 \lambda_i^\beta \lambda_n^\alpha + \sigma(\lambda_i^{\alpha+\beta} \lambda_n^\alpha + \lambda_i^\beta \lambda_n^{\alpha+\beta}) < \sigma^2 \lambda_i^\alpha \lambda_n^\beta + \sigma(\lambda_i^{\alpha+\beta} \lambda_n^\beta + \lambda_i^\alpha \lambda_n^{\alpha+\beta})$$

$$\Leftarrow \{ \begin{array}{c} \sigma^2 \lambda_i^\beta \lambda_n^\alpha < \sigma^2 \lambda_i^\alpha \lambda_n^\beta \\ \sigma(\lambda_i^{\alpha+\beta} \lambda_n^\alpha + \lambda_i^\beta \lambda_n^{\alpha+\beta}) < \sigma(\lambda_i^{\alpha+\beta} \lambda_n^\beta + \lambda_i^\alpha \lambda_n^{\alpha+\beta}) \end{array}, \ when \ \ 0 < \lambda_i \le \lambda_n \le 1, \ \ \alpha > \beta$$

Proof (ii): here, we assume that $h(\lambda) = \frac{\lambda^c}{\sigma + \lambda^c}$, then:

$\frac{dh''(\lambda)}{dc} = \frac{\lambda^{c-2}}{(\sigma + \lambda^c)^4}(\sigma M + (c^2 + c)\lambda^{2c} \ln \lambda - (2c+1)\lambda^{2c})$,here $M = (2c-1)\sigma + (c^2 - c)\sigma \ln \lambda - 2\lambda^c - 4c^2 \lambda^c \ln \lambda$.

If σ is small enough, it's easy to validate that: $\frac{dh''(\lambda)}{dc} < 0$, hence, if $\alpha > \beta$, then $(\frac{\lambda^\alpha}{\sigma + \lambda^\alpha})'' < (\frac{\lambda^\beta}{\sigma + \lambda^\beta})'' < 0$, and according to this results, we will have:

$\frac{(\lambda^\alpha / (\sigma + \lambda^\alpha))''}{\sum_{i=1}^n \lambda_i^\alpha / (\sigma + \lambda_i^\alpha)} < \frac{(\lambda^\alpha / (\sigma + \lambda^\alpha))''}{\sum_{i=1}^n \lambda_i^\beta / (\sigma + \lambda_i^\beta)} < \frac{(\lambda^\beta / (\sigma + \lambda^\beta))''}{\sum_{i=1}^n \lambda_i^\beta / (\sigma + \lambda_i^\beta)}$, that is, $f''(\lambda) < g''(\lambda)$.

Combining above results and Lemma 3, that is, if σ is enough small, then $f(\lambda)$ and $g(\lambda)$ have one and only common solution in $\lambda \in [\lambda_1, \lambda_n]$. Besides, it's easy to validate that $f(\lambda)$ and $g(\lambda)$ are both increasing functions. Here, we assumed that $\bar{\lambda}$ indicates the common solution, and $\lambda_{k-1} \le \bar{\lambda} \le \lambda_k$. Then, combining above facts, we could easily prove following conclusion: if $\lambda \in \{\lambda_k, ..., \lambda_n\}$, then $f(\lambda) > g(\lambda)$; if $\lambda \in \{\lambda_1, ..., \lambda_{k-1}\}$, then $f(\lambda) < g(\lambda)$. In fact, this conclusion is the mathematical form of result regarding effect of eigenvalue exponent on RLS-KP.

Theorem 1.0: If the regularization parameter σ is fixed, for RLS or SLP algorithm with eigenvalue transformation applied, when eigenvalue exponent $\alpha = 0$, objective function could achieve minimal value.

Proof: Firstly, we give a proof for RLS algorithm with eigenvalue transformation applied. Similar to RLS algorithm, the objective function of RLS algorithm with eigenvalue transformation applied is as follows:

$$
\begin{aligned}
J &= (vec(Y) - K^{\alpha}c)^T(vec(Y) - K^{\alpha}c) + \sigma c^T K^{\alpha} c \\
&= (VV^T vec(Y) - V\Lambda^{\alpha} V^T c)^T (VV^T vec(Y) - V\Lambda^{\alpha} V^T c) + \sigma c^T V\Lambda^{\alpha} V^T c \\
&= (V^T vec(Y) - \Lambda^{\alpha} V^T c)^T (V^T vec(Y) - \Lambda^{\alpha} V^T c) + \sigma c^T V\Lambda^{\alpha} V^T c \\
&= (vec(\tilde{Y}) - \Lambda^{\alpha} \tilde{c})^T (V^T vec(\tilde{Y}) - \Lambda^{\alpha} V^T \tilde{c}) + \sigma \tilde{c}^T \Lambda^{\alpha} \tilde{c} \\
&= \sum (\tilde{y}_i - \lambda_i^{\alpha} \tilde{c}_i)^2 + \sigma \sum \lambda_i^{\alpha} \tilde{c}_i^2 \qquad (13)
\end{aligned}
$$

Here $vec(\tilde{Y}) = V^T vec(Y)$; $\tilde{c} = V^T c$. $\tilde{y}_i$ is the i-th row element of $vec(\tilde{Y})$, $\tilde{c}_i$ is the i-th row element of $\tilde{c}$, and λ_i is the i-th diagonal element of Λ. The above second equality follows due to the reason as: $K^{\alpha} = V\Lambda^{\alpha} V^T$ and $VV^T = I$. The above third equality follows due to the reason as: $V^T V = I$. When the objective function J achieves minimal value, according to equation (13), we will have:

$$
\frac{\partial J}{\partial \tilde{c}_i} = -2(\tilde{y}_i - \lambda_i^{\alpha} \tilde{c}_i)\lambda_i^{\alpha} + 2\sigma \lambda_i^{\alpha} \tilde{c}_i = 0 \Rightarrow \tilde{c}_i = \frac{\tilde{y}_i}{\lambda_i^{\alpha} + 2\sigma} \qquad (14) .
$$

On the other hand, according to equation (13), by taking the first derivative of α, we will have:

$$
\begin{aligned}
\frac{\partial J}{\partial \alpha} &= -2\sum (\tilde{y}_i - \lambda_i^{\alpha} \tilde{c}_i)\tilde{c}_i \lambda_i^{\alpha} \ln(\lambda_i) + \sigma \sum \tilde{c}_i^2 \lambda_i^{\alpha} \ln(\lambda_i) \\
&= -3\sigma \sum \tilde{c}_i^2 \lambda_i^{\alpha} \ln(\lambda_i) \qquad (15)
\end{aligned}
$$

The above second equality follows due to equality (14). On the other hand, according to **Lemma 1**, $\lambda_i \le 1$. Therefore we will have: $-3\sigma \sum \tilde{c}_i^2 \lambda_i^{\alpha} \ln(\lambda_i) \ge 0$, that is, $\frac{\partial J}{\partial \alpha} \ge 0$. Hence, if $\alpha = 0$ and $\tilde{c}_i = \frac{\tilde{y}_i}{1 + 2\sigma}$, then the objective function J will achieve minimal value.

For SLP with eigenvalue transformation applied, the proof procedure is similar to RLS with eigenvalue transformation applied. The objective function J of SLP with eigenvalue transformation applied is as follows:

$$
\begin{aligned}
J &= \frac{\sigma}{2} vec(\bar{Y})^T (I - K^{\alpha}) vec(\bar{Y}) + \frac{1}{2} (vec(Y) - vec(\bar{Y}))^T (vec(Y) - vec(\bar{Y})) \\
&= \frac{\sigma}{2} vec(\bar{Y})^T (VV^T - V\Lambda^{\alpha} V^T) vec(\bar{Y}) + \frac{1}{2} (vec(Y) - vec(\bar{Y}))^T (vec(Y) - vec(\bar{Y})) \\
&= \frac{\sigma}{2} vec(\hat{Y})^T (I - \Lambda^{\alpha}) vec(\hat{Y}) + \frac{1}{2} (vec(\tilde{Y}) - vec(\hat{Y}))^T (vec(\tilde{Y}) - vec(\hat{Y})) \\
&= \frac{\sigma}{2} \sum \hat{y}_i^2 (1 - \lambda_i^{\alpha}) + \frac{1}{2} \sum (\tilde{y}_i - \hat{y}_i)^2 \qquad (16)
\end{aligned}
$$

Here $vec(\hat{Y}) = V^T vec(\bar{Y})$; $vec(\tilde{Y}) = V^T vec(Y)$. $\hat{y}_i$ is the i-th row element of $vec(\hat{Y})$

and $\tilde{y}_i$ is the i-th row element of $vec(\tilde{Y})$. According to equality (16), then we will have:

$$\frac{\partial J}{\partial \hat{y}_i} = \sigma(1 - \lambda_i^{\alpha})\hat{y}_i + \hat{y}_i - \tilde{y}_i = 0 \Rightarrow \hat{y}_i = \frac{\tilde{y}_i}{1 + \sigma(1 - \lambda_i^{\alpha})} \qquad (17)$$

$$\frac{\partial J}{\partial \alpha} = -\frac{\sigma}{2}\sum \hat{y}_i^2(1 - \lambda_i^{\alpha})\lambda_i^{\alpha} \ln(\lambda_i) \qquad (18)$$

According to **Lemma 1,** we will also have: $\frac{\partial J}{\partial \alpha} \geq 0$. Hence, if $\alpha = 0$ and $\hat{y}_i = \frac{\tilde{y}_i}{1 + \sigma}$, then the objective function J will achieve minimal value.

Table S1. Performance of RLS-KP by 10-fold cross validation with $S_d = S_{ATC}$

Fold Id	Original algorithm				Algorithm with eigenvalue transformation applied					
	Training set		Test set		Training set		Test set			
	AUC	AUPR	AUC	AUPR	AUC	AUPR	AUC	AUPR	α	p
1	93.7	51.1	93.8	53.4	92.2	55.3	92.0	58.7	0.7	0.002
2	93.6	51.0	93.8	56.4	92.3	55.4	92.6	62.7	0.7	0.000
3	93.4	50.4	94.2	56.4	92.4	55.1	92.5	61.2	0.7	0.012
4	93.4	50.5	95.0	58.5	93.0	55.2	93.4	63.3	0.8	0.007
5	93.3	50.8	93.8	54.7	93.0	55.1	92.9	60.2	0.8	0.000
6	93.4	50.9	93.9	54.0	92.7	55.4	92.6	58.3	0.8	0.000
7	93.9	50.3	93.7	57.8	92.9	55.2	93.0	63.3	0.7	0.000
8	93.9	50.7	94.0	58.5	92.6	54.2	93.7	63.5	0.8	0.000
9	93.9	50.5	91.8	55.9	92.8	54.5	91.0	59.6	0.8	0.000
10	93.5	50.3	95.4	58.4	92.2	54.4	94.8	63.5	0.8	0.000
average	93.6	50.6	93.9	56.4	92.6	55.0	92.8	61.4		0.002

The AUC scores and AUPR scores are normalized to 100. The p indicates p-value of bootstrapping.

Table S2. Performance of RLS-KP by 10-fold cross validation with $S_d = \frac{S_{chem} + S_{ATC}}{2}$

Fold Id	Original algorithm				Algorithm with eigenvalue transformation applied					
	Training set		Test set		Training set		Test set			
	AUC	AUPR	AUC	AUPR	AUC	AUPR	AUC	AUPR	α	p
1	94.1	42.1	94.4	45.6	94.5	60.8	94.5	67.8	0.5	0
2	94.0	41.2	95.3	49.7	94.3	60.0	96.0	71.5	0.5	0
3	94.0	41.9	94.4	49.1	94.1	61.3	94.9	69.0	0.4	0
4	94.0	41.5	95.6	47.7	94.2	60.3	96.7	70.6	0.5	0
5	94.0	41.9	93.9	46.3	94.2	61.0	93.9	68.3	0.4	0
6	93.8	41.8	94.5	46.6	94.3	60.9	95.3	70.6	0.5	0
7	94.1	41.8	93.8	44.8	94.6	61.2	94.6	67.2	0.5	0
8	94.0	41.6	93.8	47.0	94.1	61.6	93.5	66.1	0.4	0
9	94.1	41.1	94.3	46.8	94.4	60.5	94.4	69.5	0.5	0
10	94.0	41.9	92.8	44.5	94.3	61.8	93.5	67.1	0.4	0
average	94.0	41.7	94.3	46.8	94.3	60.9	94.7	68.8		0

The AUC scores and AUPR scores are normalized to 100. The p indicates p-value of bootstrapping.

Table S3. Performance of RLS-KS by 10-fold cross validation with $S_d = S_{chem}$

Fold Id	Original algorithm				Algorithm with eigenvalue transformation applied					
	Training set		Test set		Training set		Test set			
	AUC	AUPR	AUC	AUPR	AUC	AUPR	AUC	AUPR	α	p
1	92.9	56.9	94.0	66.6	93.7	56.5	94.4	66.4	1.1	0.478
2	92.8	56.6	94.3	65.2	93.2	57.2	94.3	65.3	0.6	0.453
3	92.6	57.0	93.4	63.7	93.4	57.0	93.9	63.2	1.3	0.640
4	93.1	55.7	95.2	67.3	93.3	56.0	94.8	66.6	1.3	0.969
5	92.9	57.0	93.0	62.3	93.3	57.9	93.4	62.2	1.1	0.375
6	93.4	57.1	93.5	63.1	93.6	57.4	93.4	63.0	0.8	0.647
7	93.3	57.0	93.3	63.2	93.1	57.3	93.1	63.4	0.6	0.539
8	92.7	56.6	95.4	67.6	93.3	57.2	95.3	67.6	0.6	0.640
9	93.3	57.5	92.6	62.7	93.4	58.2	92.2	62.9	0.6	0.620
10	93.4	57.5	93.1	61.8	94.0	57.5	93.0	61.1	1.5	0.822
average	93.0	56.9	93.8	64.4	93.4	57.2	93.8	64.2		0.618

The AUC scores and AUPR scores are normalized to 100. The p indicates p-value of bootstrapping.

Table S4. Performance of RLS-KS by 10-fold cross validation with $S_d = S_{ATC}$

Fold Id	Original algorithm				Algorithm with eigenvalue transformation applied					
	Training set		Test set		Training set		Test set			
	AUC	AUPR	AUC	AUPR	AUC	AUPR	AUC	AUPR	α	p
1	82.9	39.2	82.8	46.7	92.7	53.2	92.3	61.8	2	0
2	85.4	38.8	86.2	48.6	91.7	53.1	92.0	64.2	1.9	0
3	85.4	39.6	84.4	43.9	91.6	54.0	92.4	59.1	2	0
4	82.8	40.8	85.7	45.8	91.5	54.0	91.8	60.7	1.9	0
5	88.0	38.9	90.3	46.1	92.3	53.6	93.2	62.3	1.9	0
6	82.5	40.3	88.0	45.8	92.7	54.3	92.5	61.8	2	0
7	83.2	41.5	87.5	47.5	92.3	54.8	92.5	60.9	2	0
8	86.6	40.9	88.3	43.9	91.4	54.4	93.3	60.9	2	0
9	83.9	40.5	86.5	38.0	92.8	54.1	93.1	58.3	2	0
10	88.6	39.9	84.4	46.2	92.7	54.4	92.7	61.0	2	0
average	84.9	40.0	86.4	45.3	92.2	54.0	92.6	61.1		0

The AUC scores and AUPR scores are normalized to 100. The p indicates p-value of bootstrapping.

Table S5. Performance of RLS-KS by 10-fold cross validation with $S_d = \frac{S_{chem} + S_{ATC}}{2}$

Fold Id	Original algorithm				Algorithm with eigenvalue transformation applied					
	Training set		Test set		Training set		Test set			
	AUC	AUPR	AUC	AUPR	AUC	AUPR	AUC	AUPR	α	p
1	93.9	62.3	94.3	66.8	93.9	62.3	94.3	66.8	1	1.000
2	93.9	60.9	94.9	69.3	94.2	61.4	95.0	68.6	1.3	0.871
3	94.0	61.3	94.4	70.4	94.3	61.7	94.2	70.1	1.1	0.611
4	93.9	61.8	94.8	70.1	94.3	62.0	94.8	68.9	1.4	0.993
5	94.2	62.0	93.9	69.3	94.2	62.0	93.9	69.3	1	1.000
6	93.9	61.1	94.7	73.0	94.5	61.4	94.9	72.5	1.4	0.688
7	93.5	61.3	94.7	70.0	93.9	61.8	94.4	70.0	0.9	0.709
8	94.0	62.4	93.8	67.0	94.0	62.4	93.8	67.0	1	1.000
9	93.2	60.6	96.6	74.0	93.7	60.5	96.4	74.0	1.1	0.624
10	94.2	61.9	94.8	71.1	94.2	61.9	94.8	71.1	1	1.000
average	93.9	61.6	94.7	70.1	94.1	61.7	94.6	69.8		0.850

The AUC scores and AUPR scores are normalized to 100. The p indicates p-value of bootstrapping.

Table S6. Performance of RLS-avg by 10-fold cross validation with $S_d = S_{chem}$

Fold Id	Original algorithm				Algorithm with eigenvalue transformation applied					
	Training set		Test set		Training set		Test set			
	AUC	AUPR	AUC	AUPR	AUC	AUPR	AUC	AUPR	α	p
1	93.6	58.8	94.0	65.5	93.6	58.8	94.0	65.5	1	1.000
2	93.7	58.0	91.3	63.8	93.9	58.4	91.9	63.7	1.1	0.270
3	93.6	58.7	94.1	63.3	93.9	58.4	94.6	62.7	1.2	0.610
4	93.5	58.4	95.8	70.1	93.7	58.6	96.5	70.1	1.1	0.418
5	93.6	58.2	94.7	65.3	94.0	58.8	94.3	65.1	0.9	0.885
6	93.6	58.7	94.3	65.0	93.9	59.0	94.4	64.9	1.1	0.397
7	93.7	59.4	93.9	62.2	93.9	59.6	94.4	62.2	1.1	0.228
8	93.9	57.9	93.9	68.0	93.9	57.9	93.9	68.0	1	1.000
9	93.6	58.3	94.1	65.8	93.8	58.2	93.8	65.8	1.1	0.616
10	93.6	57.2	95.7	68.2	94.1	57.2	95.9	67.5	1.2	0.960
average	93.7	58.4	94.2	65.7	93.9	58.5	94.4	65.6		0.638

The AUC scores and AUPR scores are normalized to 100. The p indicates p-value of bootstrapping.

Table S7. Performance of RLS-avg by 10-fold cross validation with $S_d = S_{ATC}$

Fold Id	Original algorithm				Algorithm with eigenvalue transformation applied					
	Training set		Test set		Training set		Test set			
	AUC	AUPR	AUC	AUPR	AUC	AUPR	AUC	AUPR	α	p
1	89.6	54.6	91.7	59.6	92.7	55.6	94.4	59.2	1.4	0.037
2	89.5	54.9	92.3	60.0	93.0	55.1	93.4	59.9	1.4	0.11
3	89.1	53.8	91.7	62.9	92.2	55.5	92.4	63.9	1.1	0.009
4	89.2	54.5	92.6	62.4	91.7	55.1	93.2	63.2	1.2	0.006
5	90.1	55.3	92.5	60.2	92.0	56.3	93.2	60.2	1.3	0.123
6	90.0	53.8	90.6	61.9	92.8	54.8	92.8	61.0	1.4	0.154
7	91.5	55.4	90.2	59.8	93.3	55.6	92.9	58.9	1.5	0.066
8	91.4	53.9	91.9	64.4	92.3	54.5	94.1	64.0	1.4	0.111
9	90.1	54.0	91.7	64.3	93.5	54.1	93.4	64.5	1.5	0.061
10	90.6	54.2	92.2	65.7	92.9	54.7	93.6	67.2	1.3	0.001
average	90.1	54.4	91.7	62.1	92.6	55.1	93.4	62.2		0.0678

The AUC scores and AUPR scores are normalized to 100. The p indicates p-value of bootstrapping.

Table S8. Performance of RLS-avg by 10-fold cross validation with $S_d = \frac{S_{chem} + S_{ATC}}{2}$

Fold Id	Original algorithm				Algorithm with eigenvalue transformation applied					
	Training set		Test set		Training set		Test set			
	AUC	AUPR	AUC	AUPR	AUC	AUPR	AUC	AUPR	α	p
1	94.2	60.7	95.0	70.3	94.3	61.0	94.9	70.5	0.9	0.456
2	94.4	60.5	95.5	69.7	94.3	60.8	95.7	69.7	0.9	0.399
3	94.3	60.6	93.7	67.8	94.3	60.6	93.7	67.8	1	1.000
4	94.3	61.3	95.6	68.6	95.1	60.5	95.7	67.7	1.2	0.915
5	94.5	60.5	93.7	68.0	94.6	60.6	93.6	68.1	0.8	0.572
6	94.7	60.5	94.4	68.9	94.7	60.5	94.4	68.9	1	1.000
7	94.3	61.8	94.9	66.3	94.4	61.8	94.8	66.3	0.9	0.603
8	94.4	60.7	94.2	66.9	94.4	61.1	93.9	66.8	0.8	0.667
9	94.2	60.7	94.4	69.6	94.8	60.8	94.9	69.2	1.1	0.742
10	94.4	60.0	94.6	69.6	94.4	60.2	95.1	69.8	0.9	0.396
average	94.4	60.7	94.6	68.6	94.5	60.8	94.7	68.5		0.675

The AUC scores and AUPR scores are normalized to 100. The p indicates p-value of bootstrapping.

Table S9. Performance of SLP-KP by 10-fold cross validation with $S_d = S_{chem}$

Fold Id	Original algorithm				Algorithm with eigenvalue transformation applied					
	Training set		Test set		Training set		Test set			
	AUC	AUPR	AUC	AUPR	AUC	AUPR	AUC	AUPR	α	p
1	86.3	22.0	84.9	21.1	93.5	53.7	92.0	58.0	0.2	0
2	86.3	22.1	84.1	20.0	93.1	54.5	92.0	56.7	0.1	0
3	85.9	21.5	85.0	22.2	92.9	53.8	92.6	57.4	0.1	0
4	86.1	22.2	85.3	26.4	92.9	53.5	93.5	60.7	0.1	0
5	85.9	21.6	86.3	24.1	93.1	53.4	92.5	60.1	0.1	0
6	85.8	21.5	88.2	26.8	92.8	53.2	94.5	63.5	0.1	0
7	85.9	21.2	87.1	25.5	92.8	53.4	93.9	61.4	0.1	0
8	85.9	22.3	86.5	22.8	93.0	53.2	94.2	60.8	0.1	0
9	86.0	21.5	86.2	25.7	92.8	53.3	93.6	61.2	0.1	0
10	85.8	21.4	89.1	28.3	92.9	53.4	94.3	63.9	0.1	0
average	86.0	21.7	86.3	24.3	93.0	53.6	93.3	60.4		0

The AUC scores and AUPR scores are normalized to 100. The p indicates p-value of bootstrapping.

Table S10. Performance of SLP-KP by 10-fold cross validation with $S_d = S_{ATC}$

Fold Id	Original algorithm				Algorithm with eigenvalue transformation applied					
	Training set		Test set		Training set		Test set			
	AUC	AUPR	AUC	AUPR	AUC	AUPR	AUC	AUPR	α	p
1	47.6	12.0	46.2	10.5	74.8	20.2	46.2	10.6	0.2	0.040
2	48.0	12.4	48.9	13.3	74.5	19.8	48.9	13.4	0.3	0.198
3	47.6	11.8	48.9	13.3	77.2	15.5	49.0	13.4	0.1	0.039
4	48.7	12.7	48.1	12.7	78.7	14.8	48.0	13.1	0.3	0.312
5	49.3	12.9	48.6	12.3	51.2	13.7	48.6	12.3	0.8	0.614
6	47.8	12.1	48.2	11.8	79.5	14.2	48.2	11.9	0.1	0.069
7	49.3	12.3	50.1	13.2	49.6	12.5	50.1	13.2	1.5	0.924
8	49.7	13.1	47.7	13.0	51.3	14.4	47.7	13.0	1.2	0.770
9	48.6	12.2	49.2	14.0	50.3	12.5	49.2	14.0	0.9	0.595
10	48.6	12.5	49.5	15.2	50.9	13.7	49.5	15.2	0.9	0.700
average	48.5	12.4	48.5	12.9	63.8	15.1	48.5	13.0		0.426

The AUC scores and AUPR scores are normalized to 100. The p indicates p-value of bootstrapping.

Table S11. Performance of SLP-KP by 10-fold cross validation with $S_d = \frac{S_{chem} + S_{ATC}}{2}$

Fold Id	Original algorithm				Algorithm with eigenvalue transformation applied					
	Training set		Test set		Training set		Test set			
	AUC	AUPR	AUC	AUPR	AUC	AUPR	AUC	AUPR	α	p
1	86.9	21.9	88.2	24.4	94.1	55.9	95.2	67.8	0.1	0
2	86.9	22.5	87.3	21.1	94.2	57.5	95.0	65.5	0.1	0
3	86.9	22.4	88.4	23.5	94.1	56.9	95.6	67.0	0.1	0
4	87.1	22.3	84.7	24.6	94.0	57.4	94.0	63.1	0.1	0
5	86.8	21.7	88.5	25.6	94.4	56.8	94.2	64.9	0.1	0
6	87.0	21.0	87.3	29.0	94.1	55.7	94.5	64.9	0.1	0
7	87.0	22.5	86.9	21.3	94.1	57.5	94.6	60.3	0.1	0
8	87.3	22.6	85.1	22.4	94.3	57.8	92.4	60.6	0.1	0
9	87.0	21.9	87.0	24.9	94.1	57.4	95.4	62.3	0.1	0
10	86.8	21.0	88.0	28.7	93.7	55.5	95.1	65.4	0.1	0
average	87.0	22.0	87.1	24.5	94.1	56.8	94.6	64.2		0

The AUC scores and AUPR scores are normalized to 100. The p indicates p-value of bootstrapping.

Table S12. Performance of SLP-KS by 10-fold cross validation with $S_d = S_{chem}$

Fold Id	Original algorithm				Algorithm with eigenvalue transformation applied					
	Training set		Test set		Training set		Test set			
	AUC	AUPR	AUC	AUPR	AUC	AUPR	AUC	AUPR	α	p
1	92.2	46.8	91.6	51.1	93.4	55.7	93.2	63.1	0.1	0
2	92.1	46.7	93.4	54.1	93.4	56.1	94.8	63.9	0.1	0
3	92.2	47.0	93.0	56.3	93.5	55.4	94.7	66.1	0.1	0
4	92.0	47.2	92.8	54.0	93.6	56.2	93.8	63.2	0.1	0
5	92.2	47.8	92.6	49.0	93.6	56.6	93.4	60.2	0.1	0
6	92.0	47.3	92.8	51.6	93.1	55.8	93.7	63.2	0.1	0
7	92.1	46.5	93.0	54.1	93.7	55.1	93.7	64.6	0.1	0
8	92.2	47.7	91.6	52.3	93.1	56.6	92.4	61.9	0.1	0
9	92.0	46.7	92.0	52.3	93.4	56.3	93.9	62.6	0.1	0
10	92.2	46.9	92.4	52.5	93.4	55.4	93.7	61.8	0.2	0
average	92.1	47.1	92.5	52.7	93.4	55.9	93.7	63.1		0

The AUC scores and AUPR scores are normalized to 100. The p indicates p-value of bootstrapping.

Table S13. Performance of SLP-KS by 10-fold cross validation with $S_d = S_{ATC}$

Fold Id	Original algorithm				Algorithm with eigenvalue transformation applied					
	Training set		Test set		Training set		Test set			
	AUC	AUPR	AUC	AUPR	AUC	AUPR	AUC	AUPR	α	p
1	49.70	12.60	47.70	12.50	75.00	17.80	47.70	12.50	0.4	0.804
2	65.2	18.0	48.9	15.7	76.9	13.2	48.9	15.7	1.2	0.797
3	75.7	14.4	50.8	16.3	75.7	14.4	50.8	16.3	1	1.000
4	50.2	13.6	49.5	14.0	74.2	16.4	49.6	14.0	0.7	0.472
5	62.1	20.5	49.3	13.0	79.1	15.0	49.3	12.8	0.2	0.679
6	49.1	12.1	51.6	15.3	63.5	14.9	51.6	15.2	1.6	0.737
7	50.3	13.9	51.1	16.4	67.9	21.7	51.1	16.4	0.5	0.749
8	68.4	17.0	46.1	10.1	77.5	15.3	46.3	10.2	0.2	0.462
9	48.3	12.2	66.0	19.8	73.8	15.4	66.5	20.8	0.9	0.421
10	73.0	15.6	50.2	12.7	76.4	14.6	50.1	12.7	0.6	0.681
average	59.2	15.0	51.1	14.6	74.0	15.9	51.2	14.7		0.680

The AUC scores and AUPR scores are normalized to 100. The p indicates p-value of bootstrapping.

Table S14. Performance of SLP-KS by 10-fold cross validation with $S_d = \frac{S_{chem} + S_{ATC}}{2}$

Fold Id	Original algorithm				Algorithm with eigenvalue transformation applied					
	Training set		Test set		Training set		Test set			
	AUC	AUPR	AUC	AUPR	AUC	AUPR	AUC	AUPR	α	p
1	93.0	45.5	94.0	52.4	93.8	58.7	95.1	66.6	0.1	0
2	93.4	45.6	92.5	54.1	94.4	58.8	93.6	67.9	0.1	0
3	93.0	46.6	93.1	45.9	94.1	60.4	94.6	63.2	0.1	0
4	93.2	46.5	93.4	48.9	94.2	59.2	94.7	64.5	0.1	0
5	93.1	46.1	92.6	48.0	94.0	58.4	94.3	63.0	0.1	0
6	92.9	45.5	93.0	53.0	94.2	58.4	94.2	67.8	0.1	0
7	93.1	45.9	93.5	51.8	94.0	59.0	94.4	65.9	0.1	0
8	92.9	45.6	94.0	53.5	94.3	58.4	94.3	67.3	0.1	0
9	93.1	45.3	93.4	53.1	93.9	58.3	94.9	67.3	0.1	0
10	93.0	46.0	93.3	49.5	94.2	58.9	94.4	64.6	0.1	0
average	93.1	45.9	93.3	51.0	94.1	58.9	94.4	65.8		0

The AUC scores and AUPR scores are normalized to 100. The p indicates p-value of bootstrapping.

Table S15. Performance of SLP-avg by 10-fold cross validation with $S_d = S_{chem}$

Fold Id	Original algorithm				Algorithm with eigenvalue transformation applied					
	Training set		Test set		Training set		Test set			
	AUC	AUPR	AUC	AUPR	AUC	AUPR	AUC	AUPR	α	p
1	92.3	46.4	91.9	51.5	93.7	55.0	93.1	60.6	0.2	0
2	92.1	47.6	93.3	51.7	93.4	56.9	94.1	62.8	0.2	0
3	91.9	45.9	92.7	57.9	93.2	54.5	94.1	67.7	0.2	0
4	92.0	46.3	92.8	55.5	93.5	55.3	94.2	66.7	0.2	0
5	92.3	47.7	92.2	52.9	93.5	56.0	93.0	62.2	0.2	0
6	91.9	47.0	93.9	54.5	93.1	56.0	94.8	64.4	0.2	0
7	92.2	47.6	90.7	48.0	93.2	56.0	92.5	58.7	0.2	0
8	92.2	47.1	91.6	50.9	93.1	55.8	93.0	60.5	0.2	0
9	92.3	46.8	92.6	52.7	93.4	55.6	94.1	63.4	0.2	0
10	92.0	47.6	92.9	51.9	92.9	56.0	94.3	61.6	0.2	0
average	92.1	47.0	92.5	52.7	93.3	55.7	93.7	62.9		0

The AUC scores and AUPR scores are normalized to 100. The p indicates p-value of bootstrapping.

Table S16. Performance of SLP-avg by 10-fold cross validation with $S_d = S_{ATC}$

Fold Id	Original algorithm				Algorithm with eigenvalue transformation applied					
	Training set		Test set		Training set		Test set			
	AUC	AUPR	AUC	AUPR	AUC	AUPR	AUC	AUPR	α	p
1	93.1	41.4	94.0	41.3	92.2	50.8	94.3	53.8	0.4	0
2	92.9	40.6	94.6	49.0	93.1	49.9	94.0	59.2	0.5	0
3	93.0	41.0	92.9	46.4	92.2	51.4	91.0	58.5	0.3	0
4	93.0	40.8	94.5	45.8	92.8	50.2	94.1	56.8	0.5	0
5	93.3	41.4	92.2	44.5	92.7	51.1	91.7	55.4	0.4	0
6	93.0	40.5	93.5	46.3	92.4	50.6	93.1	57.1	0.4	0
7	93.0	40.2	94.0	50.2	92.2	49.7	93.8	60.8	0.4	0
8	93.3	40.7	93.3	46.4	92.5	50.7	93.0	57.9	0.4	0
9	93.2	41.9	92.4	43.6	92.2	52.0	91.8	55.7	0.4	0
10	93.0	40.8	93.8	42.7	92.0	50.9	92.4	53.8	0.4	0
average	93.1	40.9	93.5	45.6	92.4	50.7	92.9	56.9		0

The AUC scores and AUPR scores are normalized to 100. The p indicates p-value of bootstrapping.

Table S17. Performance of SLP-avg by 10-fold cross validation with $S_d = \frac{S_{chem} + S_{ATC}}{2}$

Fold Id	Original algorithm				Algorithm with eigenvalue transformation applied					
	Training set		Test set		Training set		Test set			
	AUC	AUPR	AUC	AUPR	AUC	AUPR	AUC	AUPR	α	p
1	92.8	45.9	93.1	52.5	94.0	58.2	94.2	65.7	0.1	0
2	93.1	46.3	92.2	46.9	94.0	59.0	93.2	61.9	0.1	0
3	93.0	46.2	93.5	51.5	94.2	58.3	94.6	67.0	0.1	0
4	93.2	46.1	92.6	48.3	94.4	58.8	93.8	63.1	0.1	0
5	93.1	46.0	93.3	50.2	94.1	58.4	94.5	65.2	0.1	0
6	93.1	45.7	94.1	52.4	94.1	58.3	95.3	67.5	0.1	0
7	93.2	46.2	92.1	48.7	94.4	58.5	93.4	63.1	0.1	0
8	93.2	45.6	93.3	53.5	94.5	58.0	94.6	65.6	0.1	0
9	93.0	45.2	94.2	57.7	93.9	57.4	95.4	70.0	0.1	0
10	93.0	45.8	94.0	49.1	94.2	57.4	95.4	62.6	0.2	0
average	93.1	45.9	93.2	51.1	94.2	58.2	94.4	65.2		0

The AUC scores and AUPR scores are normalized to 100. The p indicates p-value of bootstrapping.
